# Immune Checkpoint Inhibitor Associated Autoimmune Encephalitis, Rare and Novel Topic of Neuroimmunology: A Case Report and Review of the Literature

**DOI:** 10.3390/brainsci12060773

**Published:** 2022-06-13

**Authors:** Yining Gao, Jie Pan, Dingding Shen, Lisheng Peng, Zhifeng Mao, Chunxia Wang, Huanyu Meng, Qinming Zhou, Sheng Chen

**Affiliations:** 1 Department of Neurology, Ruijin Hospital, Shanghai Jiao Tong University School of Medicine, Shanghai 201801, China; gynsjtu@163.com (Y.G.); dds@ntu.edu.cn (D.S.); mengh_y@126.com (H.M.); 2 Department of Neurology, The First Hospital of Jiaxing & The Affiliated Hospital of Jiaxing University, Jiaxing 314001, China; jxyypj@126.com; 3 Co-innovation Center of Neuroregeneration, Nantong University, Nantong 226019, China; 4 Department of Neurology, The Third Affiliated Hospital of Sun Yat-sen University, Guangzhou 510630, China; lishengpeng79@163.com; 5 Neuroimmunology Group, KingMed Diagnostic Laboratory, Guangzhou 510310, China; maozf216@163.com; 6 Department of Clinical Medicine, Medical School, Xiangnan University, Chenzhou 423043, China; 7 The State Key Laboratory of Translational Medicine and Innovative Drug Development, Ministry of Science and Technology, Jiangsu Simcere Diagnostics Co., Ltd., No. 699-18 Xuanwu Ave, Nanjing 210002, China; chunxia.wang@simceredx.com; 8 Institute of Neurology, Shanghai Jiao Tong University, Shanghai 200240, China

**Keywords:** immune checkpoint inhibitors, immune-related adverse event, autoimmune encephalitis, pembrolizumab

## Abstract

Immune checkpoint inhibitors (ICIs) are being used in patients with various advanced malignancies, and patient outcomes have improved considerably. Although ICIs can effectively treat tumors, 30–60% of patients experience immune-related adverse events (irAEs). Autoimmune encephalitis (AE) is a rare irAE that has become a novel topic in neuroimmunology and has received increasing attention in recent years. Herein, we report a rare case of GAD65-antibody–associated AE after metastatic small cell lung cancer treatment with pembrolizumab. The patient received IVIg therapy for AE and continuous pembrolizumab therapy without suspension of tumor treatment. At 1 year follow-up, both the patient’s AE symptoms and tumors were stable. We consider that the treatment of ICI-associated AE should be more individualized with prudent decision-making and should balance the tumor progression and AE treatment. In addition, we have also comprehensively reviewed the literature of ICI-associated AE, and summarized the clinical features, treatment, and prognosis of AE caused by ICI, thus broadening our understanding of the neurological complications caused by ICI.

## 1. Introduction

Immune checkpoint inhibitors (ICIs) are a class of monoclonal antibodies that target regulatory immune checkpoint molecules that inhibit T cell activation. ICIs can enhance T cell-mediated anti-tumor immunity and promote immune-mediated tumor cell clearance by blocking co-inhibitory signaling pathways [1,2,3]. Their antitumor effects have been recognized in clinical trials and have been approved by the FDA for the treatment of malignant tumors, such as melanoma, non-small cell lung cancer, colorectal cancer, and hepatocellular carcinoma [4]. 

ICIs can be divided into monoclonal antibodies against CTLA-4 and monoclonal antibodies against PD-1/PD-L1 depending on the pathways they act on [5]. Monoclonal antibodies against PD-1/PD-L1 are the favorable method for modern immunotherapy of solid tumors [6,7,8]. ICIs can enhance the anti-tumor immune response by removing the inhibitory effect of PD-1 or PD-L1 immune checkpoints on the activation and proliferation of T cells, and reduce the number and/or inhibitory activity of Treg cells [9]. By doing so, the killing function of T cells against the tumor is restored, leading to the inhibition of tumor development [1]. However, when ICIs induce activation of the immune system, they also nonspecifically lead to the destruction of the immune homeostasis of non-tumor tissues, resulting in severe immune and inflammatory reactions. These include clinical immune-related adverse events (irAEs) [10,11]. At present, the specific mechanism of irAE generation is not clear, and it is generally believed to be related to the disorder of immune homeostasis caused by ICI treatment [12,13]. There are large amounts of ICs on the surface of non-tumor cells; ICI combined with them can lead to the activation of complement in the body and increase the level of inflammation, thereby disrupting immune homeostasis [14].

Common irAEs include skin rash, itching, colitis, hepatitis, and all types of endocrine diseases [15]. Nervous system irAEs are relatively rare, which include central nervous system (CNS) irAEs and peripheral nervous system (PNS) irAEs. PNS irAEs mainly include myasthenia gravis, Guillain–Barre syndrome, and peripheral sensory motor neuropathy [16]. CNS irAEs are much rarer than PNS irAEs. ICI-associated autoimmune encephalitis (AE) is an uncommon complication that has rarely been reported [17,18], and thus not completely understood. Herein, we report the first case of pembrolizumab-associated GAD65 antibody AE with a favorable short-term prognosis after treatment. Furthermore, in order to better understand the CNS complications caused by ICI, we comprehensively reviewed ICI-associated AE. 

## 2. Materials and Methods

References for the review were identified by searching English literature in the PubMed database published from 2016 to 2022, using the search terms (alone or in logical combinations): “immune checkpoint inhibitor”, “autoimmune encephalitis”, “immune-related adverse events”, “anti PD1”, “anti PDL1”, and “anti CTLA4.” The inclusion criteria are as follows: patients with (1) encephalitis symptoms identical to classical AE, regardless of whether neuronal autoantibodies were detected; (2) any AE symptoms associated with classic tumor neuronal autoantibodies; or (3) symptoms associated with autoantibodies against synaptic receptors or other neuron cell surface proteins after ICI treatment. Patients were excluded if neuronal autoantibodies were detected before ICI treatment [19].

## 3. Case Report

**Figure 1:**
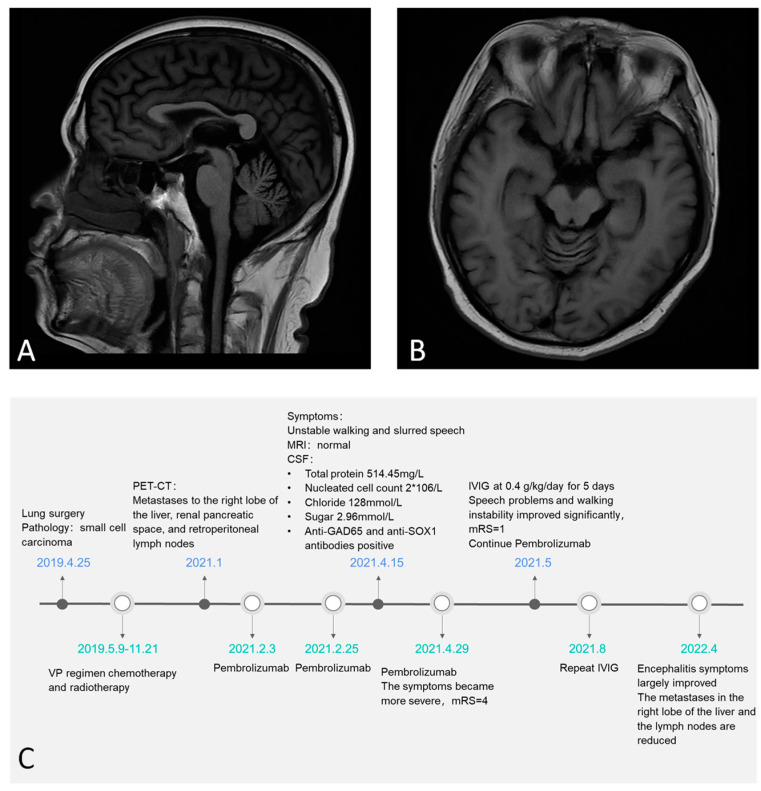
Mild cerebella atrophy evidence on sagittal and axial brain MRI (**A**,**B**). The timeline of the patient (**C**).

A 51-year-old man was admitted with an unsteady gait and slurred speech, which persisted for 20 days. He had undergone pulmonary nodule resection in April 2019. Frozen section pathology revealed small-cell carcinoma in the anterior segment of the right upper lobe. He received multiple doses of VP-16, carboplatin, chemotherapy, and radiotherapy from May 2019 to November 2020. In January 2021, positron emission tomography-computed tomography (PET-CT) revealed two round and low-density lesions with increased fluorodeoxyglucose (FDG) metabolism on the right liver lobe, enlarged lymph nodes with FDG hypermetabolism in the retroperitoneal area and the space between the right kidney and pancreas. The size of lesions on the right lobe of the liver were 1.3 × 1.1 cm and 1.34 × 1.44 cm. Considering the liver and lymph node metastases, on the 3rd and 25th of February 2021, the patient received pembrolizumab therapy. On 15 April 2021, he developed slurred speech and an unsteady gait. The patient was again treated with pembrolizumab on the 29 of April 2021, and his symptoms progressed significantly and his modified Rankin Scale (mRS) score deteriorated to 4. Neurological examination revealed dysmetria on the finger-to-nose test, positive Romberg’s sign, ataxic gait, and nystagmus. The cranial nerves were intact and there was no nuchal rigidity. The patient had normal muscle strength, muscle tone, and tendon reflexes. Cerebrospinal fluid (CSF) analysis showed normal opening pressure (120 mmH_2_O), nucleated cell count 2 × 10^6^/L, protein 514.45 mg/L, chloride 128 mmol/L, and sugar 2.96 mmol/L. Cranial magnetic resonance imaging (MRI) showed evidence of mild cerebellar atrophy (Figure 1A,B). ^18^F-FDG-PET-CT indicated decreased metabolism in both cerebellar hemispheres. 

The autoimmune encephalitis antibody panel revealed that anti-GAD65 and anti-SOX1 antibodies were present in both serum and CSF, while other antibodies were absent. The titers of GAD65 and SOX1 antibodies in the serum were 1:30 and 1:10, respectively; and while they were 1:100 and 1:10, respectively, in the CSF. Therefore, the patient was diagnosed with pembrolizumab-associated AE. The irAE grade was 3 according to the National Cancer Institute Common Terminology Criteria for Adverse Events version 5.0 (CTCAE-5.0) [20]. The patient was given intravenous immunoglobulin (IVIg) at 0.4 g/kg/day for 5 days, and his speech and gait improved significantly. His mRS improved to 1. Considering that excessive immunosuppressive therapy might lead to tumor recurrence, this patient did not receive glucocorticoid or immunosuppressive therapy. Furthermore, pembrolizumab treatment was continued for the tumor. He received IVIg therapy 3 months later to prevent the recurrence of AE symptoms. At the 1-year follow-up, his AE symptoms had largely recovered, and the tumor was stable. The titer of GAD65 antibodies in serum decreased to 1:1. CT showed that the size of the metastases in the right lobe of the liver were 0.3 × 0.51 cm and 0.67 × 0.88 cm. The retroperitoneal lymph nodes and lymph nodes in the right renal space between the pancreas were also reduced (Figure 1C, timeline for this patient). 

## 4. Discussion

### 4.1. Pembrolizumab-Associated GAD65 Antibody AE, a Very Rare Complication of ICI

The incidence of irAEs in the nervous system after ICI treatment is 2% to 6% [21,22], of which the incidence of encephalitis is 0.05% [16]. As far as we know, ICI-associated AE is currently limited to a single case report or a few small case series [18,23]. Cases of ICI-associated AE with autoantibodies are very rare. This is the first case of pembrolizumab-associated GAD65 antibody-positive AE. We noticed that this patient experienced a reduction in tumor size and progression of ataxia with GAD65 and SOX1 autoantibodies after pembrolizumab treatment, which indicated a diagnosis of pembrolizumab-associated AE [19]. In this patient, pembrolizumab was very effective in the treatment of tumors; both the tumor size in the lung and tumor metastasis were well controlled after the treatment. In addition, after five days of IVIg treatment, the patient’s AE symptoms improved significantly, and we resumed the pembrolizumab treatment. At 1-year follow-up, both the patient’s AE symptoms and tumors were stable. In previous reports, steroids rather than IVIg were generally chosen for ICI-associated AE [24], and ICI treatment was usually discontinued. However, in our patient, timely and effective immunotherapy and continued treatment with pembrolizumab resulted in significant reduction of both AE and tumors. Our case may provide new directions for future treatment of ICI-associated AE. 

### 4.2. Literature Review of ICI-Associated AE

By reviewing the literature, we found 50 cases of ICI-associated AE; of which, 28 and 22 cases were with and without autoantibodies, respectively. Patients’ age ranged from 19 to 81 years, with a mean age of 61.5 years, with a male-to-female ratio of 1:1. The patients had lung cancer (20), melanoma (12), renal cancer (6), pleural mesothelioma (3), Hodgkin’s lymphoma (2), ovarian cancer (1), breast cancer (1), endometrial cancer (1), uterine cancer (1), Merkel cell carcinoma (1), thymic carcinoma (1), and urothelial carcinoma (1). Thirty-nine patients were explicitly prescribed with ICI-specific drugs: Nivolumab (15), ipilimumab/Nivolumab (11), Pembrolizumab (7), Sintilimab (1), Durvalumab (2), Dostarlimab (1), and Atezolizumab (2). After the use of ICI, the patients presented with AE. The time from ICI use to AE symptom onset ranged from 4 days to 18 months (median, 3 months). Examination of CSF showed increased an cell number in 21 patients, increased protein in 35 patients, and a positive oligoclonal band (OCB) in 7 patients. Twenty-eight patients were found to have positive autoimmune antibodies in the cerebrospinal fluid or serum, including Ma2 Ab (10), GAD Ab (7), Hu Ab (3), NMDAR Ab (4), SOX1 Ab (2), Ri Ab (1), GABAbR Ab (1), and CASPR2 Ab (1) [25]. Hu Ab and NMDAR Ab were double-positive in one patient [26]. According to CTCAE-5.0, there were 3 Grade 2 cases, 29 Grade 3 cases, and 15 Grade 4 cases [20]. After treatment with steroids, IVIg, plasma exchange, and rituximab, the symptoms of encephalitis improved in 31 patients; 13 patients did not improve, and 6 patients died (Table 1).

This table summarized the reported cases of ICI-associated AE (abbreviations: NA = not available, FLAIR = fluid-attenuated inversion recovery, MTL = mesial temporal lobe, OCB = oligoclonal band). We noticed that among the reported cases, only seven cases were related to the GAD65 antibody [51], ranging in age from 33 to 64 years. All patients developed neuropsychiatric symptoms after using nivolumab alone or in combination with ipilimumab; none have been reported for pembrolizumab with the GAD65 antibody. Among the seven patients, four had poor prognosis or died. Six patients suspended ICI immediately after the onset of AE symptoms. Piepgras et al. reported a patient whose treatment with nivolumab was continued after controlling symptoms with steroids and infliximab but died after the encephalitis symptoms recurred [36]. In contrast to the previously reported patient, our patient had a good prognosis at the 1-year follow-up. The prognosis of ICI-associated AE varied after treatment.

The mechanisms of ICI-associated AE may be due to the following reasons: (1) The immune response induced by ICI may cross-react with CNS autoantigens; (2) AE is closely associated with tumors [52]. ICI may simultaneously enhance the immune response of the tumor and the immune response to CNS; and (3) ICI may recognize innate immune molecules on the surface of neurons, and thus directly kill neurons through the complement system or cytotoxicity, leading to the release of intracellular antigens [53].

In addition to AE, ICI can also lead to other neurological complications. Neurological syndromes caused by ICI are mainly peripheral neuropathies, including myasthenia gravis [54], Guillain–Barre syndrome [55], chronic polyneuropathies [56], and mononeuropathies [57]. CNS complications include noninfectious encephalitis, demyelinating disease, and cerebral artery vasculitis [16]. Neurological complications associated with ICI treatment require medical attention.

### 4.3. Current Dilemma: The Balance between ICI Therapy, Tumor Progression, and AE Treatment

Immune-related adverse events are classified into five grades [20]. Due to the presence of pathogenic autoantibodies, the treatment of ICI-associated AE includes withdrawal of ICI, IVIg, plasma exchange, and immunosuppressive therapies. As discussed above, most scholars support stopping ICI therapy and initiating immunosuppressive therapy [58]. According to the principle of toxicity management, only Grade 1 irAEs could continue ICI. Grades 2–4 irAEs should withhold ICI and receive stronger immunosuppressive therapy [59]. However, discontinuation of ICI therapy may lead to tumor progression. In addition, strong immunosuppression is associated with tumor recurrence. Being able to balance the treatments may prove difficult. Based on our experience, the treatment of ICI-associated AE should be more individualized and prudent. We advocate that IVIg or plasma exchange may be the first choices for Grade 2 and Grade 3 patients, and ICI may not be discontinued at first. The patients need more close monitoring. If patients do not respond to first-line treatment or symptoms progression, ICI treatment needs to be suspended and stronger immunosuppressants can be used [60]. In the literature we reviewed, we found two Grade 2 patients who continued using ICI or were switched to another type of ICI, and had good prognosis [24]. However, because ICI-associated AE is a relatively new topic in neuroimmunology, we need more cases and multi-center research in the future. 

## 5. Conclusions

This is the first case of pembrolizumab-associated AE with GAD65 and SOX1 antibodies, characterized by progressive cerebellar ataxia. It should be noted that this patient received IVIg therapy for AE and continuous pembrolizumab therapy without suspension of tumor treatment. At present, the treatment of ICI-associated AE needs to be more individualized with close monitoring, evaluation, and prudent decision-making. Finally, the mechanisms, clinical features, and treatment of ICI-associated AE are a new concept in the field of neuroimmunology, and further studies are needed.

## Figures and Tables

**Table 1:** ICI-associated AE.

Author	ICI	Age	Sex	Onset Time	Cancer	Antibody (CSF/Serum)	Symptoms and irAEs Grade	CSF (White Cells/Protein/OCB) and MRI	Treatment	Prognosis
Brown [27]	Pembrolizumab	67	M	7 M	Melanoma	CASPR2 Ab(+/+)	Short-term memory loss, anxiety episodes, G3	↑/N/NA T2 hyperintensity of the MTL bilaterally	steroid	partially improved
Kopecky [28]	Nivolumab	64	M	3 M	Renal cancer	Ma2 Ab(+/−)	Uncontrollable movements, choreiform movements, G3	N/N/NA increased signal within the basal ganglia	steroid, infliximab	deceased
Shah [29]	Nivolumab	44	F	4 M	Lung adenocarcinoma	GAD Ab(+/+)	Dysarthria, dyskinesias, refractory seizures, G4	↑/N/NA T2 signal hyperintensities of the bilateral MTL compatible with limbic encephalitis	ICIs discontinued, steroid, PLEX, rituximab	partially improved
	Nivolumab	66	F	4 M	Lung cancer		Dysarthria, dysarthric, dyskinesias, bilateral arm and leg ballismus, G3	N/↑/NA symmetric T2 hyperintense and T1 hypointense basal ganglia abnormalities	ICIs discontinued, steroid, IVIg, rituximab	deceased
Williams [17]	Ipilimumab/Nivolumab	56	F	18 D	Melanoma	NMDAR Ab(+/−)	Disorientation, inattention, bradykinesia, hyperreflexia, G3	↑/N/NA stable encephalomalacia at sites of prior radiosurgery with no additional metastases	ICIs discontinued, steroid, IVIg	partially improved
	Ipilimumab/Nivolumab	65	M	NA	Lung cancer	SOX1 Ab(−/+)	Short-term memory loss, progressive difficulty ambulating, G3	↑/↑/NA new nonspecific T2 hyperintensities in the right MTL	ICIs discontinued, steroid	improved
Gill [26]	Pembrolizumab	71	F	3 M	Lung adenocarcinoma	Ri Ab(+/−)	Diplopia, unsteady gait, urinary incontinence, G4	↑/↑/NA Normal	ICIs discontinued, steroid, rituximab	not improved
	Nivolumab	68	F	NA	Merkel cell carcinoma	Hu Ab(+/+), NMDAR Ab(+/−)	Progressively altered mental status, truncal ataxia, vertical nystagmus, G3	N/↑/+ T2/FLAIR hyperintensities bilaterally in the medial temporal lobes	ICIs discontinued, steroid, IVIg, rituximab	not improved
Shibaki [30]	Nivolumab	78	M	9 D	Pleural mesothelioma	Ma2 Ab(−/+)	Fever, anorexia, somnolence syndrome, nystagmus, G3	↑/↑/NA T2 high signal intensity in the mesencephalon and medial thalamus	ICIs discontinued, steroid	improved
Vogrig [23]	Pembrolizumab	79	M	2 M	Lung cancer	Ma2 Ab(NA/NA)	Impulsivity and disinhibition, hyperphagia, confusion, decreased consciousness, G4	N/↑/NA NA	ICIs discontinued, steroid	partially improved
	Ipilimumab/Nivolumab	71	M	5 M	Pleural mesothelioma	Ma2 Ab(+/+)	Narcolepsy-cataplexy, hyperphagia, psychiatric symptoms, G3	↑/↑/NA FLAIR hypersignal involving the uncus bilaterally, periventricular regions of the third ventricle and hypothalamus	ICIs discontinued, steroid, rituximab	not improved
	Nivolumab	57	F	8 M	Pleural mesothelioma	Ma2 Ab(+/+)	Memory deficits, epilepsy, psychomotor retardation, G4	NA/↑/NA FLAIR bilateral MTL hypersignal	ICIs discontinued, steroid, IVIg	not improved
	Pembrolizumab	47	M	8 M	Lung cancer	Ma2 Ab(+/+)	Ophthalmoplegia, head drop, G3	N/N/+ FLAIR bilateral MTL hypersignal	ICIs discontinued, Steroid	not improved
	Nivolumab	55	M	3 M	Kidney cancer	Ma2 Ab(+/+)	Right ear hearing loss, ataxia, vertigo, memory deficits, G3	N/↑/NA FLAIR bilateral MTL hypersignal	ICIs discontinued, steroid, PLEX	not improved
	Nivolumab	69	M	3 M	Kidney cancer	Ma2 Ab(+/−)	Confusion, focal seizures, G3	N/↑/NA FLAIR bilateral MTL hypersignal	ICIs discontinued, Steroid	not improved
Fellner [31]	Nivolumab	26	F	NA	Hodgkin lymphoma	Ma2 Ab(−/+)	Seizures, G4	N/N/NA FLAIR signal changes in MTL	ICIs discontinued, Steroid	improved
	Ipilimumab/Nivolumab	19	F	NA	Melanoma		Fever, altered mental state, G4	↑/↑/NA NA	ICIs discontinued, Steroid	improved
N. Shah [32]	Pembrolizumab	70	M	17 M	Melanoma	NMDAR Ab(+/+)	Hypoactive delirium, recurrent falls, brief witnessed tonic-clonic seizures, G4	N/↑/NA Normal	ICIs discontinued, steroid, IVIg	not improved
Chung [18]	Ipilimumab/Nivolumab	36	F	2 M	Thymoma	GAD Ab(+/N)	Progressive short-term memory loss, seizures, G4	N/N/NA fluid-attenuated inversion recovery hyperintensities involving the MTL and hippocampi bilaterally	ICIs discontinued, steroid, IVIg	deceased
Lyons [33]	Nivolumab	56	F	3.5 M	Renal cancer	Ma2 Ab(+/+)	Seizure, memory loss, behavioral and personality changes, left internuclear, ophthalmoplegia, G4	N/↑/NA multiple areas of increased T2 fluid-attenuated inversion recovery signal intensity in the temporal lobes, frontal lobes, brainstem, including bilateral limbic structures and left temporal cortex	ICIs discontinued, steroid, IVIg, mycophenolate mofetil	not improved
Kang [34]	Sintilimab	66	F	3 M	SCLC	Hu Ab(+/N)	Focal seizures, G3	↑/N/NA NA	ICIs discontinued, steroid	partially improved
Hottinger [35]	Ipilimumab/Nivolumab	71	F	4 D	SCLC	Hu Ab(+/N)	Memory deficits, G2	↑/N/NA severe abnormalities in both hippocampi with contrast-enhancing lesions	ICIs discontinued, steroid, natalizumab	partially improved
Piepgras [36]	Ipilimumab/Nivolumab	52	F	2 W	Melanoma	GAD Ab(+/+)	Short-term memory loss, cognitive dysfunction, limb ataxia, epileptic seizures, G4	↑/↑/NA small alterations	ICIs discontinued, steroid, infliximab, reuse nivolumab	deceased
Burke [37]	Nivolumab	64	F	4 M	Ovarian clear cell cancer	GAD Ab(−/+)	fever, stiff arms and legs, occasional spasms, G3	N/N/NA Normal	steroid, PLEX	partially improved
Duong [38]	Nivolumab	57	M	5 W	SCLC	GAD Ab(−/+)	NA	↑/N/NA FLAIR signal in MTL	steroid, IVIg	deceased
	Nivolumab	64	M	6 M	SCLC	SOX1 Ab(+/−)	NA	↑/N/+ NA	steroid, IVIg	deceased
	Ipilimumab/Nivolumab	71	F	18 M	NSCLC		NA	↑/↑/NA FLAIR signal in temporal lobes and thalami	steroid	partially improved
Ghous [39]	Ipilimumab/Nivolumab	33	M	1 M	Melanoma	GAD Ab(+/−)	Slurred speech, word-finding difficulty, ataxia, lower extremity hyperreflexia, G3	N/↑/+ Normal	steroid	partially improved
Maniscalco [40]	Nivolumab	63	M	3 M	Melanoma	GAD Ab(+/+)	seizures, memory loss, behavioral changes, walking difficulties, G4	↑/N/+ limbic involvement	ICIs discontinued, steroid, IVIg	not improved
Shechtman [41]	Durvalumab	66	F	2 M	SCLC	GABAbR Ab(+/+)	Seizures, disorientation, memory disturbances, G4	N/N/NA mild chronic microvascular ischemic changes	steroid	partially improved
Yordduangjun [42]	Dostarlimab	52	F	NA	Endometrial cancer	NMDAR Ab(NA/NA)	Confusion, tremors, loss of fine motor skills, G3	↑/↑/+ right temporal sclerosis	ICIs discontinued, steroid, IVIg, rituximab	partially improved
Taliansky [24]	Anti CTLA4	70	M	20 D	SCLC		Seizures, speech disturbances, G4	NA/↑/NA Normal	ICIs discontinued, NA	partially improved
	Anti PD1	87	M	12 D	Urothelial carcinoma		Confusion, G3	NA/↑/NA Normal	ICIs discontinued, NA	not improved
	Anti CTLA4+anti PD1	49	F	9 D	Uterine carcinoma		Cerebellar ataxia, opsoclonus, tremor, G3	NA/↑/NA Normal	ICIs discontinued, NA	partially improved
	Anti PD1	71	F	24 D	Breast cancer		Psychotic state, G3	NA/↑/NA Normal	ICIs discontinued, NA	partially improved
	Anti PD1	84	M	21 D	Melanoma		Confusion, somnolence, G2	NA/N/NA Normal	NA, ICIs continued	partially improved
	Anti PD1	59	M	210 D	Melanoma		Confusion, somnolence, headache, G2	NA/↑/NA Normal	NA, change the ICI type	partially improved
	Anti PD1	71	F	110 D	NSCLC		speech and behavioral disturbance, generalized and complex partial epileptic event, G3	NA/↑/NA Abnormal	ICIs discontinued, NA	not improved
	Anti PD1	68	M	150 D	NSCLC, adenocarcinoma		Confusion, generalized epileptic event, G4	NA/↑/NA Normal	ICIs discontinued, NA	partially improved
	Anti PD1	67	F	15 D	NSCLC, adenocarcinoma		Confusion, sensory neuropathy, G3	NA/↑/NA Normal	ICIs discontinued, NA	partially improved
	Anti PD1+anti LAG3	67	F	11 D	Melanoma		Ataxia, speech disturbances, partial seizure, G3	NA/↑/NA Normal	ICIs discontinued, NA	partially improved
	Anti PD1	73	F	15 D	Renal cancer		Headache, confusion, G3	NA/↑/NA Normal	ICIs discontinued, NA	partially improved
Yamaguchi [43]	Atezolizumab	56	M	17 D	Lung cancer		Consciousness disturbance, motor aphasia, G3	↑/↑/NA Normal	ICIs discontinued, steroid	partially improved
QUACH [44]	Pembrolizumab	69	M	2 M	Melanoma		Headaches, fever, and altered mental status, G3	N/↑/NA suspected left vertebral artery occlusion, known cavernous venous malformation, chronic right optic nerve atrophy	ICIs discontinued, steroid	improved
Braden [45]	Ipilimumab/Nivolumab	61	M	7 M	Melanoma		Sudden onset aphasia, left lower limb myoclonic jerks, confusion, G3	N/↑/NA FLAIR hyperintensity in the right MTL with differentials including encephalitis or postictal changes	ICIs discontinued, steroid	partially improved
Nishijima [46]	Atezolizumab	72	F	7 M	NSCLC		Gait disturbance, mild disturbance of consciousness, G3	N/N/+ symmetrical high signal in the thalamus bilaterally	steroid, IVIg	not improved
Shionoya [47]	Durvalumab	68	F	1 M	SCLC		disorientation, memory impairment, eating difficulty, G3	↑/↑/NA Normal	ICIs discontinued, Steroid	improved
NIKI [48]	Pembrolizumab	51	M	6 M	NSCLC		seizure, difficulty in walking and communicating, G4	↑/↑/NA a tumor in the right frontal lobe of the brain	ICIs discontinued, Steroid	partially improved
Nalbantoğlu [49]	Nivolumab	40	M	1 M	Hodgkin lymphoma		disorientation, inattention, postural tremor in the upper left extremity, and ataxia G3	↑/↑/+ right occipital, left frontal millimetric lesions with gadolinium enhancement	ICIs discontinued, Steroid	partially improved
Thouvenin [50]	Ipilimumab/Nivolumab	70	M	3 W	Renal cancer		Confusion, gait disturbance, aphasia, G3	↑/↑/NA MRI was limited because of the patient’s agitation	ICIs discontinued, Steroid	partially improved

## Data Availability

The data presented in this study are available on request from the corresponding author. The data are not publicly available due to the patient privacy.
